# The production of β-glucosidases by *Fusarium proliferatum* NBRC109045 isolated from Vietnamese forest

**DOI:** 10.1186/2191-0855-2-49

**Published:** 2012-09-14

**Authors:** Ziqing Gao, Duong Van Hop, Le Thi Hoang Yen, Katsuhiko Ando, Shuichi Hiyamuta, Ryuichiro Kondo

**Affiliations:** 1 Department of Agro-Environmental Sciences, Faculty of Agriculture, Kyushu University, Fukuoka, Japan; 2 Center of Biotechnology, Vietnam National University, Hanoi, Vietnam; 3 Department of Biotechnology, National Institute of Technology and Evaluation, Tokyo, Japan; 4 Advanced Technology Laboratories IDEMITSU KOSAN Co., Ltd, Chiba, Japan

**Keywords:** *Fusarium proliferatum*, β-glucosidases, Differential expression, The translation elongation factor 1-α

## Abstract

*Fusarium proliferatum* NBRC109045 is a filamentous fungus isolated from Vietnamese forest due to high production of β-glucosidases. Production of the enzyme was studied on varied carbon source based mediums. The highest activity was obtained in medium containing 1% corn stover + 1% wheat bran (3.31 ± 0.14 U/ml). It is interesting to note that glucose (0.69 ± 0.02 U/ml) gave higher activity and just followed by cellobiose among the di- and mono-saccharides, which is generally regarded as a universal repressor of hydrolases. We improved the zymogram method to prove that in response to various carbon sources, *F. proliferatum* could express various β-glucosidases. One of the β-glucosidases produced by *F. proliferatum* growing in corn stover + wheat bran based medium was partially purified and proved to have high catalytic ability.

## Introduction

Biofuels derived from lignocellulosic biomass are emerging as promising alternatives to fossil fuels to meet the increasing global energy demands (Ragauskas et al. 2006). One of the key steps in bioconversion process is the enzymatic hydrolysis of the cellulose polymers in the biomass to monomeric sugars that are subsequently fermented to ethanol (Percival et al. 2006; Adsul et al. 2007). The three main categories of players in cellulose hydrolysis are cellobiohydrolases (or exo-1, 4-β-glucanases) (EC 3.2.1.91), endo-1, 4-β-glucanases (EC 3.2.1.4), and β-glucosidases (EC 3.2.1.21) (Beguin and Aubert 1994). The endo-1, 4-β-glucanases randomly attack cellulose in amorphous zones and release oligomers. The cellobiohydrolases liberate cellobiose from reducing and non-reducing ends. And finally β-glucosidases hydrolyze the cellobiose and in some cases the cellooligosaccharides to glucose (Ryu 1980; Wood 1985). Cellulose polymers are degraded to glucose through sequential and cooperative actions of these enzymes. Cellobiohydrolases and endoglucanases are often inhibited by cellobiose, making β-glucosidases important in terms of avoiding decreased hydrolysis rates of cellulose over time due to cellobiose accumulation (Workman and Day 1982). Low efficiency and high costs associated with the enzymatic hydrolysis process present a major bottleneck in the production of ethanol from lignocellulosic feedstocks (Banerjee et al. 2010). For the enzymatic conversion of biomass to fermentative sugar on a commercial scale, it is necessary to have all cellulolytic components at the optimal level. Since β-glucosidases activity is low in many microbial preparations used usually for the saccharification process (Enari 1983). It is necessary to supply additional β-glucosidases to such reaction. In order to optimize the use of different biomasses, it is important to identify new β-glucosidases with improved abilities on the specific biomasses as well as with improved abilities such as stability and high conversion rates. β-Glucosidases have potential roles in various fields such as the food, pharmacology and cosmetic industries and also in the valorisation of some products, due to the properties of this enzyme to convert and to synthesize biomolecules of high added value (Esen 1993). There are hundreds of different β-glucosidic flavor precursors in plants, and their hydrolysis often enhances the quality of the beverages and foods produced from them (Gϋnata 2003; Esen 2003). Aside from flavor enhancement, foods, feeds, and beverages may be improved nutritionally by release of vitamins, antioxidants, and other beneficial compounds from their glycosides (Opassiri et al. 2004). Indeed, β-glucosidase can either degrade or synthesize small carbohydrate polymers, depending on particular experimental conditions (Crout and Vic 1998). The β-glucosidases can be arranged in three groups related to localization: intracellular, cell wall associated, and extracellular. Primarily the extracellular β-glucosidases are of industrial interest (Soewnsen 2010). The number of fungal species on earth is estimated to 1.5 million of which as little as approximately 5% are known (Hawksworth 1991; 2001). So there is a statement that calls for all-out effort to unravel the potential of unknown species found in nature. The identification and characterization of new fungal species are often encountered in literature. Cuc Phuong Park and Ba Be Park is the old national one in Vietnam and boasts an engaging cultural and wildlife heritage and enchanting scenery. Covered in a dense forest, these landscapes are rich and diverse tropical and subtropical species of microorganisms for wood and plant degradation. In the present study, a potential β-glucosidases-producing fungus NBRC109045 was isolated from Ba Be national park and identified as *Fusarium proliferatum*. Under optimized conditions, *F. proliferatum* produces β-glucosidases with an activity of 3.3 U/ml based on *p*NPG as substrate and an activity of 426 U/ml based on cellobiose as substrate. In this paper, we described ways that (a) isolating and screening microbes to produce considerable quantities of β-glucosidases; (b) modifying the method of zymogram to prove that different carbon sources direct varied β-glucosidases expression in *F. proliferatum*; (c) assaying partial purification to prove high catalytic efficiency of β-glucosidase produced by *F. proliferatum* growing in corn stover + wheat bran based medium.

## Materials and methods

### Materials

Unless specified otherwise, all chemicals were of analytical grade. Solubilized crystalline cellulose was obtained from Kyokuto Seiyaku Co., Ltd, Japan. Avicel [(R) RH-101], 4-methylumbelliferyl-β-D-glucoside (MUG) and carboxymethyl cellulose (CMC) were products of Sigma Chemical Co., (St. Louis, Mo, USA). Cellobiose, xylose, glucose, sucrose, galactose and maltose were purchased from Wako Pure Chemical Industries, Ltd, Japan. 4-Nitrophenyl-β-D-glucopyranoside monohydrate (*p*NPG) was purchased from Tokyo Chemical Industry Co., Ltd, Japan. Corn stover was collected from Yingkou city, Liaoning Province in China. Wheat bran and bagasse were obtained from private companies.

### Strains isolation

Wood chip of *Jatropha carcass*, branch and leaves of *J. carcass*, wood chip of *Manihot esculenta*, branch and leaves of *M*. *esculenta*, coconut shell, sugarcane, and rice straw were used as lignocellulosic sources for degradation in Vietnamese National Park (Ba Be and Cuc Phuong). One month later, lignocellulosic sources were dug up. All strains that would be screened were isolated from degraded biomass samples and washed soil collected. Isolated strains were inoculated on solubilized crystalline cellulose (CC) plates and CMC plates to cultivate for two weeks (Deguchi et al. 2007). The microbes that could grow on CC and CMC were picked up and inoculated onto malt extract agar (MEA).

### Screening of β-glucosidases-producing strains

#### The first step of screening

For primary screening, strains from MEA were plated on potato dextrose agar (PDA) medium in a 9-cm diameter Petri dish and incubated at 30°C for 5 days. Then the colonies were inoculated on β-glucosidases (EC 3.2.1.21) screening agar containing 1% of CMC, 0.5% of MUG, 1.5% of agar, and Mandels salts (Daenen et al. 2008). The cultures were incubated at 30°C for 3 days. Then the plates were observed under UV light. Colonies which showed fluorescence were sorted out. It is because methylumbelliferyl (MU) which was released from MUG by β-glucosidases can emit fluorescence when induced by UV light.

#### The second step of screening

For secondary screening, the mycelium of the β-glucosidases-producing isolates obtained from the primary screening was transferred to a new PDA medium in a 9-cm diameter Petri dish and incubated at 30°C. Once the fungus covered most of the PDA plate, agar plates with mycelium were transferred to a sterile blender containing 25 ml of sterile water and homogenized for 30 s. Ten ml of the fungal homogenate was used to inoculate into β-glucosidases secondary screening medium containing 1% corn stover + 1% wheat bran in 100 ml, pH 5.0 Mandels salts medium with KH_2_PO_4_ 2 g l^-1^, (NH_4_)_2_SO_4_ 1.4 g l^-1^, urea 0.69 g l^-1^, CaCl_2_·2H_2_O 0.3 g l^-1^, MgSO_4_·7H_2_O 0.3 g l^-1^, and 1 ml trace elements solution composing of MnSO_4_ 1.6 g l^-1^, ZnSO_4_ 2 g l^-1^, CuSO_4_ 0.5 g l^-1^, CoSO_4_ 0.5 g l^-1^ (Saibi et al. 2011) then incubated at 30°C, 150 rpm for 5 days. Crude enzyme extract was obtained by centrifuging the liquid medium at 20 000 g, 4°C for 20 min and collecting the supernatant for confirming the β-glucosidases activity.

### Enzyme assay

β-Glucosidases activity towards *p*-nitrophenyl-β-D-glucopyranoside (*p*NPG) was measured with use of amount of *p*-nitrophenol (*p*NP) liberated from *p*NPG by using a calibration curve at 410 nm (Cai et al. 1998). The reaction mixture contained 0.5 ml, 2 mM *p*NPG in 50 mM sodium acetate buffer (pH 5.0) and an appropriately diluted enzyme solution 0.125 ml. After incubation at 45°C for 10 min, the reaction was stopped after adding 1.25 mL, 1 M Na_2_CO_3,_ and the color that formed as a result of *p*NP liberation was measured at 410 nm. One unit of β-glucosidases activity was defined as the amount of enzyme required to liberate 1 μmol of *p*NP per minute under the assay conditions. Specific activity is defined as the number of units per milligram of protein.

Cellobiase activity was assayed using cellobiose as substrate. The enzymatic reaction mixtures (1 ml) containing 0.25 ml of enzyme solution and 0.75 ml of 0.5% cellobiose in 50 mM sodium acetate buffer (pH 5.0) were incubated for 30 min at 50 C. And then the mixtures were heated at 100 C for 5 min to stop the reaction. The amount of glucose released was measured by Bio-sensor (Oji Scientific Instruments Co., Itd). One enzyme unit was defined as the amount of enzyme that produced 1 μmol of glucose per minute.

### Protein concentration determination

Protein concentrations in the enzyme preparations were determined with application of the method of Bradford (Bradford 1976) with reference to a standard calibration curve for bovine serum albumin (BSA).

### Strain identification

#### DNA extraction and PCR amplification from cultures

Mycelia cultured on malt extract agar were harvested with a spatula, and DNA was extracted with use of a PrepMan® Ultra Reagent (Life Technologies, Carlsbad, California, USA). ITS-5.8S rDNA (ITS) and the D1/D2 regions of LSU rDNA (LSU) were amplified with the KOD FX (Toyobo, Osaka, Japan), and with primers ITS5 (GGAAGTAAAAGTCGTAACAAGG) and NL4 (GGTCCGTGTTTCAAGACGG) (O'Donnell 1993; White et al. 1990). The mixture was processed by following the manufacturer’s instructions of kit. The DNA fragments were amplified in a T-gradient thermal-cycler (Biometra, Göttingen, Germany). Thermal-cycling program for LSU and ITS was: initial denaturation at 94°C for 2 min, 30 cycles of denaturation at 98°C for 10 s, annealing at 56°C for 30 s, extension at 68°C for 1 min and a 4°C soak. Amplified DNA was purified with use of the Agencourt® AMPure® Kit (Agencourt Bioscience, Beverly, Massachusetts, USA).

#### DNA sequencing

Sequencing reactions were performed with the BigDye® Terminator 3.1 Cycle Sequencing Kit (Applied Biosystems, Foster City, California, USA), and with primers NL1 (GCATATCAATAAGCGGAGGAAAAG) and NL4 (GGTCCGTGTTTCAAGACGG) for LSU on the T-gradient thermal-cycler (Biometra). This thermal-cycler program was employed: initial denaturation at 96°C for 1.5 min, 35 cycles of denaturation at 96°C for 10 s, annealing at 50°C for 5 s, extension at 60°C for 1.5 min and a 4°C soak. Sequencing reaction products were purified with the Agencourt® CleanSEQ® Kit (Agencourt Bioscience) and sequenced with the ABI PRISM® 3730 Genetic Analyzer (Applied Biosystems). Contiguous sequences were assembled with ATGC software (Genetyx, Tokyo, Japan).

#### Phylogenetic analysis

DNA was analyzed with use of CLUSTAL W (Thompson et al. 1994). Based on the EF-1α sequence of *Fusarium* genus (O'Donnell et al. 2012), phylogenetic tree was generated with use of the neighbor-joining algorithm in the MEGA ver5.0. Concordance of the EF-1a gene datasets was evaluated with the partition-homogeneity test implemented with MEGA (Tamura et al. 2011), using 1 000 random repartitions. The fungus was determined to be most closely related to *Fusarium proliferatum* by comparing it with related strains in GenBank. And the NBRC deposition number is NBRC109045.

#### Effect of different carbon sources on β-glucosidases production by *F. proliferatum*

The mycelium stored on PDA medium was transferred to new PDA medium in 9-cm diameter Petri dish and incubated at 30°C for 5 days. Once the fungus covered most of the PDA plate, agar plates with mycelium were transferred to a sterile blender containing 25 ml of sterile water and then homogenized for 30 s. Ten ml of the fungal homogenate was used to inoculate 100 ml of liquid pre-cultures, pH 7.0. Liquid pre-cultures were made according to the modified Mandels medium with and without 0.69 g L^-1^ urea supplemented with 0.1% of yeast extract and 1% of glucose (Saibi et al. 2011). After 3 days, the mycelium homogenate made by a sterile blender was used to inoculate the modified Mandels medium which containing 2% carbon source with and without urea as following, wheat bran, corn stover, 1% wheat bran + 1% corn stover, bagasse, CMC, Avicel cellulose, sucrose, cellobiose, glucose, xylose, galactose and maltose. β-Glucosidases production by *F. proliferatum* in shaking flask batch cultures was carried out at 30°C and 150 rpm. Samples were withdrawn at different times during 12 days, and then centrifuged at 20 000 g for 20 min. Supernatants as crude enzyme were assayed for β-glucosidases activity, determined for pH, and analyzed by zymogram. Each culture was carried out in triplicate.

#### Electrophoresis and zymogram

Zymography is an electrophoretic technique for detection of purified or partly purified β-glucosidase. Zymography is based on SDS-PAGE that includes a substrate such as MUG or *p*NPG, which can be degraded by β-glucosidases. The degradation product emits fluorescence or produces change of color during the reaction period. However, this is not a practical method to assay β-glucosidases existing in the crude enzyme because various β-glucosidases existing in the crude enzyme caused overlapping fluorescence bands. A modified method that combines effective isolation with identification was developed to overcome the limitation of zymogram in the application on crude enzyme.

Step1: add the loading buffer for SDS-PAGE to the crude enzyme solution that was produced by incubating *F. proliferatum* in corn sotver + wheat bran based medium and glucose based medium, but the mix was not heated at a temperature of 100°C (Laemmli 1970). The mix of the crude enzyme and loading buffer was injected into the gel. Each sample was injected into four different wells and then the electrophoresis was applied.

Step2: After the electrophoresis, the first column of each sample was cut out of the gel and then treated with Coomassie Brilliant Blue (CBB) staining. The remaining gel was soaked in 20 mM, pH8.5 Tris–HCl buffer for two hours in order to remove SDS, so that the activity can be regained. The buffer was replaced every 30 min.

Step3: The first column that had been treated with CBB staining was used as a marker to cut the protein bands of the second column. The protein bands cut out of the second column were soaked in 20 mM *p*NPG for 10 min at a temperature of 45°C with the aim of active staining, and then 1.25 ml of 1 M Na_2_CO_3_ solution were added. If the color of the bands changes from colorlessness to yellow, it means that β-glucosidases exist in the bands.

Step4: Corresponding bands were cut out of the third and the fourth column based on positions of active bands of the second column. The cuts containing β-glucosidases were soaked in acetate buffer (0.05 M, pH5.0), and were crushed and separated by centrifugation. The supernatant was taken out and mixed with the same volume of loading buffer and then was analyzed with SDS-PAGE. Protein was stained with silver stainIIkit (Wako Pure Chemical Industries, Ltd, Japan).

#### Partial purification of β-glucosidase

Fine and dried powder of ammonium sulfate was added, over ice, into the crude extract enzyme to 50% saturation. And then the mix was still stirring at 4°C for 30 min. After centrifugation (42 500 g, 60 min), supernatant was decanted and the precipitate was discarded. Ammonium sulfate was added to bring the supernatant to 80% saturation. The latter was stirred overnight at 4°C and then centrifuged again. The precipitate was dissolved and dialyzed against 20 mM Tris–HCl buffer (pH 8.5). The dialyzed enzyme solution was centrifuged to remove the insoluble component and applied on the DEAE sepharose CL-6B column (1.5*20 cm) equilibrated with 20 mM Tris–HCl buffer (pH 8.5). The nonadsorbed protein fraction was eluted from the column with starting buffer (100 mL), and the adsorbed enzyme was collected through 5-stepwise elution chromatography (sodium chloride concentration: 0.1 M, 0.15 M, 0.2 M, 0.25 M and 0.3 M in the same buffer). There are two active peaks eluted from DEAE-Sepharose CL-6B at about 0.15 M and 0.25 M NaCl. The active fractions (0.15 M NaCl) were pooled and concentrated by a Centrifugal Filter Devices (Millipore Corporation Billerica, MA, USA), and then chromatographed separately on a superdex 75 column (1.5*60 cm) equilibrated with 20 mM Tris–HCl buffer (pH 8.5). The proteins were eluted with the same buffer at a flow rate of 1 mL min^-1^.

## Results

### Screening of β-glucosidases-producing strain

MUG released MU when MUG was catalyzed by β-glucosidases, and MU emitted fluorescence. In order to screen the best strain for β-glucosidases production, firstly the potential strains were cultivated in medium that contained MUG. Of these potential strains, 4 strains showed the brightest fluorescence (Table 1). Next, these 4 strains were prepared in a medium that contained 1% of corn stover and 1% of wheat bran for five days. Of these 4 strains, SIID 11460 showed the highest activity of β-glucosidases. Therefore, SIID 11460 was selected for further research.

**Table 1:** Screening of microorganism with β-glucosidases production

**No.**	**Serial number**	**Sample site**	**Source**	**Fluorescence remarks**
81	SIID11445	Cuc Phuong National Park	*Manihot esculenta *wood chip	+++
82	SIID11446	Cuc Phuong National Park	Rice straw	+
83	SIID11447	Ba Be National Park	*Manihot esculenta *wood chip	+
84	SIID11448	Cuc Phuong National Park	Soil around plant chip	+
85	SIID11449	Cuc Phuong National Park	Soil around plant chip	++
86	SIID11450	Cuc Phuong National Park	Soil around plant chip	++
87	SIID11451	Cuc Phuong National Park	*Manihot esculenta *wood chip	+
88	SIID11452	Cuc Phuong National Park	*Jatropha carcass *wood chip	+
89	SIID11453	Ba Be National Park	*Jatropha carcass *wood chip	+
90	SIID11454	Ba Be National Park	*Jatropha carcass *stems and leaves	+++
91	SIID11455	Cuc Phuong National Park	*Jatropha carcass *stems and leaves	++
92	SIID11456	Ba Be National Park	Soil around plant chip	+++
93	SIID11457	Ba Be National Park	*Jatropha carcass *wood chip	+
94	SIID11458	Ba Be National Park	*Jatropha carcass *wood chip	++
95	SIID11459	Ba Be National Park	Rice straw	+
96	SIID11460	Ba Be National Park	Coconut	+++
97	SIID11461	Ba Be National Park	Rice straw	+
98	SIID11462	Cuc Phuong National Park	Coconut	+

+++: with the brightest fluorescence.

++: with brighter fluorescence.

+: with bright fluorescence.

### Strain identification

**Figure 1:**
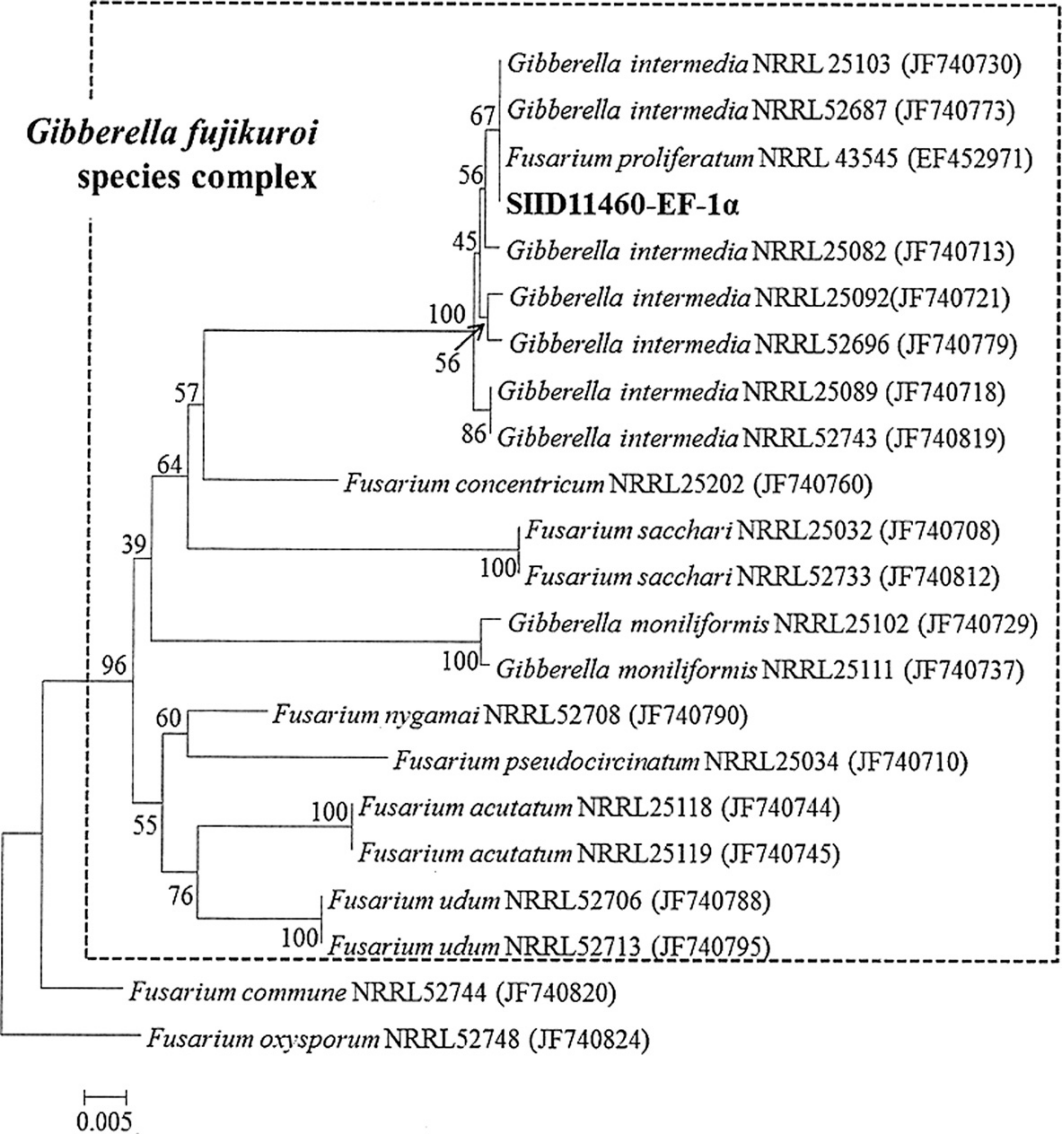
**Phylogenetic tree based on EF-1α sequences of isolated strain SIID 11460 and other related species obtained from NCBI. **The phylogenetic tree was constructed by the neighbor-joining method using CLUSTAL W and MEGA ver5.0. Levels of bootstrap support were indicated at nodes. The scale bar represents 0.005 nucleotide substitution per position.

The ITS1-5.8-ITS2 ribosomal RNA gene of SIID11460 was amplified with PCR for identification. However, amplification showed no significant differences among the sequences of the PCR products generated with the internal transcribed spacer (ITS) primers. Due to many fusaria within the *Gibberella* clade possess non-orthologous copies of ITS2, it can lead to incorrect phylogenetic inferences with use of ITS sequence identification (O'Donnell and Cigelnil 1997; O'Donnell et al. 1998). Therefore, the elongation factor 1α (EF-1α) was used for the identification of SIID11460. The EF-1α gene of SIID11460 was successfully amplified by PCR. The fungal EF-1α gene was amplified from genomic DNA, and then purified, sequenced and analyzed with the BLAST program from NBRC. The strain showed the highest identity (99.3 ~ 100%) with *Gibberella intermedia* (*Fusarium proliferatum*). Based on the EF-1α sequence of *Fusarium* genus (O'Donnell et al. 2012), phylogenetic tree was built up. Phylogenetic analysis indicated that SIID11460 and *Gibberella intermedia* NRRL 25103, *Gibberella intermedia* NRR52687 and *Fusarium proliferatum* NRRL 43545 belong to the same clade (Figure 1). Based on the comparison of the EF-1α gene sequences and the location of clade in the species complex of *Gibberella fujikuroi* (O'Donnell et al. 1998; Nirenberg and O'Donnell 1998), the strain SIID11460 was identified as a strain of *F. proliferatum* that belongs to Liseola section of the *Fusarium* genus (Nelson et al. 1983) and its teleomorph is *Gibberella intermedia.* SIID11460 was named as *F. proliferatum*NBRC109045. 

### β-glucosidases production by *F. proliferatum* in various carbon sources

Various carbon sources, not only agricultural by-products and polysaccharides but also mono- and disaccharides were tested for β-glucosidases production by *F. proliferatum* with and without urea for 10-day cultivation (Table 2). All substances with urea addition induced β-glucosidases production at different levels. When *p*NPG was used as substrate to measure activity of β-glucosidases, the activity reached the highest level of 3.31 **±** 0.14 U/ml with use of corn stover + wheat bran as carbon source. The activity level was still as high as 2.09 ± 0.13 U/ml when wheat bran was used as carbon source. An activity of 0.69 ± 0.02 U/ml was assayed when the glucose was used as carbon source even though glucose is regarded as a universal repressor of hydrolases. The activity level produced with use of glucose as carbon source was a little bit below the activity level produced with use of cellobiose as carbon source.

**Table 2:** **The activity of β-glucosidases produced by *F. proliferatum *growing on different carbon sources**

**Carbon sources**	**With urea (U/ml)**	**Without urea (U/ml)**
**Agricultural by-products**		
Corn stover	0.90 ± 0.05	0.28 ± 0.04
Wheat bran	2.09 ± 0.13	1.26 ± 0.07
Baggase	1.32 ± 0.08	1.28 ± 0.08
Corn stover + wheat bran	3.31 ± 0.14	2.78 ± 0.10
**Polysaccharides**		
CMC	0.37 ± 0.01	0.38 ± 0.08
Avicel cellulsoe	0.2 ± 0.004	0.10 ± 0.01
**Disaccharides**		
Sucrose	0.24 ± 0.04	0
Cellobiose	0.90 ± 0.03	0
**Monosaccharides**		
Glucose	0.69 ± 0.02	0
Xylose	0.28 ± 0.08	0
Galactose	0.02 ± 0.001	0
Maltose	0.02 ± 0.002	0

β-Glucosidases activity was determined based on *p*NPG as the substrate. The different carbon sources were used at the concentration of 2% in modified Mandels culture medium. Values are means ± SD of triplicate samples.

**Figure 2:**
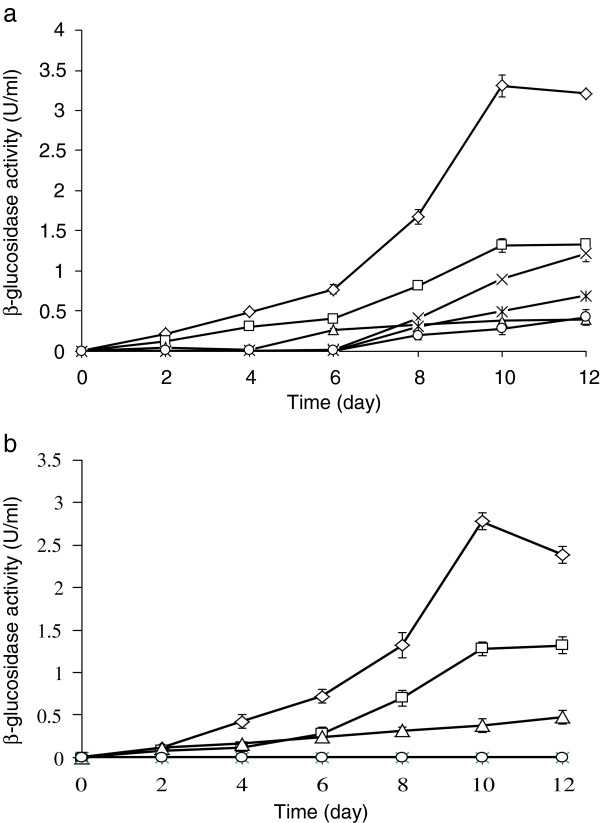
**Time course of β-glucosidases production by *F. proliferatum *using different carbon sources a: with addition of urea. b: **without addition of urea. Corn stover + wheat bran (⋄), bagasse(□), CMC(△), cellobiose(Χ), glucose(*), and xylose (○) were used individually, at the concentration of 2% in the modified Mandels medium. Samples were withdrawn every two days during 12 days.

When disaccharides and monosaccharides were used as the sole source of carbon at pH 7.0 without urea, no activity of β-glucosidase was detected even extending the period of cultivation to 25 days. Only agricultural by-products and polysaccharides at pH 7.0 without urea addition induced β-glucosidases production. The variation of pH before and after culturing was expressed in Table 3. Before cultivation of *F. proliferatum*, the pH of mediums was adjusted to 7.0. Ten days later, the pH values of glucose or cellobiose based mediums without urea addition dropped to approximately 2.5; the pH values of glucose or cellobiose based mediums with urea addition hardly changed; the pH values of corn stover + wheat bran based mediums with and without urea addition were 7.1 and 6.0, respectively, after 10-day cultivation. It is reported that the biosynthesis of β-glucosidases is greatly influenced by pH (Tangnu et al. 1981; Desrochers et al. 1981). For *F. proliferatum* in this study, low pH of the glucose or cellobiose based mediums cut production of β-glucosidases. But addition of urea halted reduction in pH of glucose or cellobiose based mediums. When *F. proliferatum* grew in corn stover + wheat bran based medium, the pH decreased slightly. Therefore, whether adding urea to corn stover + wheat bran based medium did not affect production of β-glucosidases evidently. Thus, the addition of urea might have the ability to promote the production of β-glucosidases, especially in mono and disaccharides. To make sure of the function of urea, a comparative test was carried out. Figure 2 indicated the time course of β-glucosidases production by *F. proliferatum* using different carbon sources with and without addition of urea. *F. proliferatum* started to produce β-glucosidases on the 8^th^ day after incubating in glucose or cellobiose based medium with urea addition (Figure 2-a). According to the time course for β-glucosidases production, the same amount of urea was added to the glucose and cellobiose based mediums on the 8^th^ day after incubating, respectively. Then the samples were taken out every 2 days to determine the activity of β-glucosidases and pH. However, *F. proliferatum* did not produce β-glucosidases and the pH of the mediums was kept at about 2.5. The results indicated that there was no relationship between addition of urea and halting reductions in pH of glucose or cellobiose based medium. 

**Table 3:** **The pH of mediums in which *F. proliferatum *grew for 10 days**

	**With urea addition**			**Without urea addition**		
	**Before cultivation pH**	**After cultivation pH**	**BGL activity (U/ml)**	**Before cultivation pH**	**After cultivation pH**	**BGL Activity (U/ml)**
Glucose	7.0	6.5 ± 0.2	0.69 ± 0.02	7.0	2.5 ± 0.2	0
cellobiose	7.0	6.5 ± 0.2	0.90 ± 0.03	7.0	2.6 ± 0.3	0
Corn stover + wheat bran	7.0	7.1 ± 0.1	3.31 ± 0.14	7.0	6.0 ± 0.1	2.78 ± 0.02

BGL: β-glucosidases.

**Figure 3:**
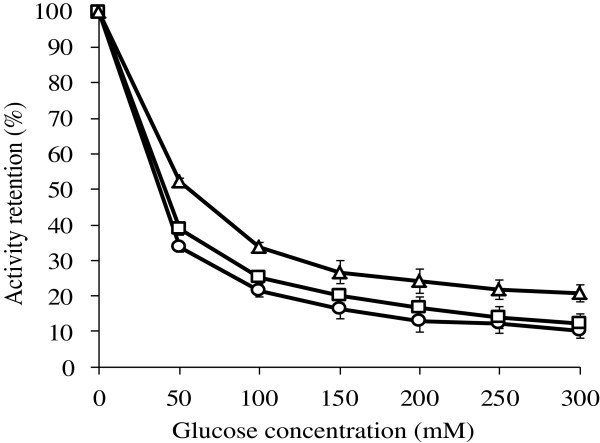
**The glucose tolerance of the β-glucosidases produced by *F. proliferatum *growing in varied carbon sources based mediums. **Corn stover + wheat bran (△), cellobiose (□), and glucose (○). Values are means ± SD of triplicate samples.

Figure 3 indicated that the glucose tolerance of the β-glucosidases produced by *F. proliferatum* growing in varied carbon sources based mediums. Supplementation of glucose in the substrate resulted in severe reductions in β-glucosidases activity. On the other hand, β-glucosidases produced by *F. proliferatum* growing in corn stover + wheat bran based medium had higher tolerance to glucose compared to that in glucose or cellobisoe based medium. β-Glucosidases produced with use of different carbon sources have different level of tolerance to the glucose.

β-Glucosidases may be classified into three groups on the basis of substrate specificity. (1) Aryl β-glucosidases exclusively hydrolysing or showing a great preference towards aryl β-glucosides; (2) cellobiases hydrolysing cellobiose and small oligosaccharides and finally (3) the members of the third group, termed as broad-specificity β-glucosidases, that act on both substrates (aryl-β-glucosides, cellobiose and cellooligosaccharides) and are the most commonly observed group in cellulolytic microbes (Patchett et al. 1987). The hydrolysis capacity of β-glucosidases produced by *F. proliferatum* growing in corn stover + wheat bran based medium and glucose based medium were tested on cellobiose (0.5%). After 30 min, aliquots were taken out and their glucose contents were determined by Bio-sensor. Based on the substrate of cellobiose, the activities of β-glucosidases produced by *F. proliferatum* growing in corn stover + wheat bran based medium and glucose based medium were 426 U/ml and 187 U/ml, respectively. According to the results mentioned above and those in Table 2, β-glucosidases produced by *F. proliferatum* grew in corn stover + wheat bran based medium and glucose based medium belongs to the third group of β-glucosidases, due to the capacity of β-glucosidases to hydrolyze cellobiose and *p*NPG.

### Differential expression of β-glucosidases in response to carbon sources

**Figure 4:**
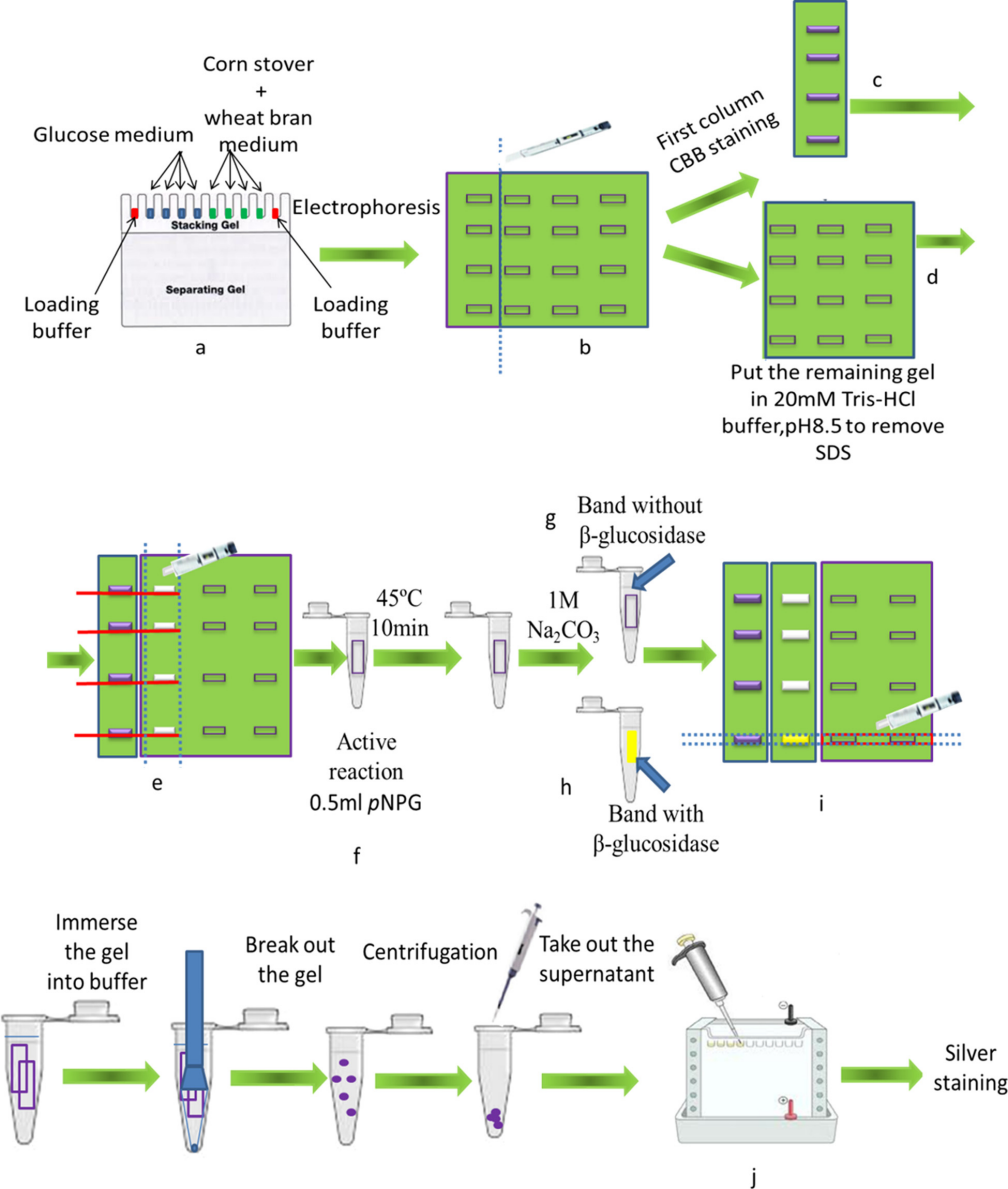
**Schematic of the modified zymogram. a: **add the mix of loading buffer and crude enzyme solution to the gel. But the mix was not heated at 100°C. Blue: crude enzyme from glucose based medium; Green: crude enzyme from corn stover + wheat bran based medium; Red: loading buffer only. **b: **after electrophoresis, the first column of each sample was cut out.** c: **the first column of each sample was stained with CBB. **d: **the remaining gel was soaked in Tris–HCl buffer to remove SDS. **e: **the first column after CBB staining was used as a marker to cut the protein bank of the second column. **f: **the protein bank cut of the second column was soaked in *p*NPG for active staining. **g: **after adding Na_2_CO_3_, the band coming from the second column kept colorlessness. **h: **the color of the band from the second column changed from colorlessness to yellow following addition of Na_2_CO_3_. **i:** according to the position of active band of the second column, cut the corresponding bands of the third and fourth column. **j: **protein coming from the bands of the third and fourth column was injected to the gel for SDS-PAGE following a series of treatments.

**Figure 5:**
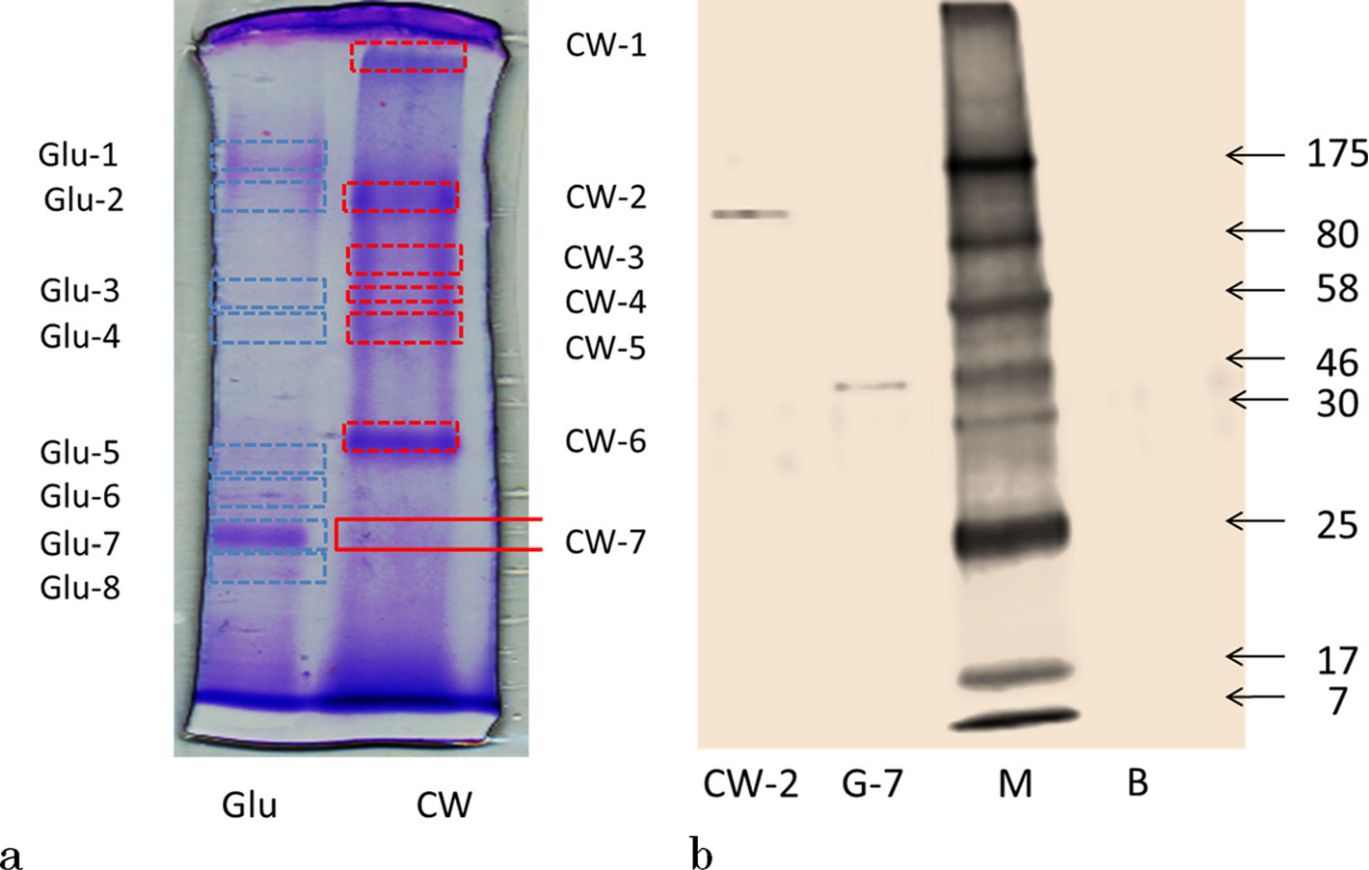
**Zymogram demonstrated that *F. proliferatum *expressed differentially in response to various carbon source at 2% (w/v) a: Coomassie staining of SDS-PAGE of crude enzyme. b**: Silver staining of the SDS–PAGE. Glu, glucose; CW, corn stover + wheat bran; M, molecular weight marker (kDa); B, loading buffer only.

Zymogram analysis was used to assay the β-glucosidases produced by *F. proliferatum* that grew in corn stover + wheat bran based medium and glucose based medium. When zymogram analysis was used to detect different β-glucosidases existing in the crude enzyme, the exact number of the fluorescence bands could not be identified because the fluorescence bands overlapped each other, and it was also difficult to get clear pictures. Therefore, we modified the zymogram method and usefully applied the modified method to prove a differential expression pattern of β-glucosidases produced by *F. proliferatum* that grew in the carbon sources (Figure 4). After the electrophoresis, the first column of each sample was cut out of the gel and then treated with Coomassie Brilliant Blue (CBB) staining. Figure 5-a shows 8 bands of proteins that existed in the crude enzyme growing in glucose based medium and 6 bands of proteins that existed in the crude enzyme growing in corn stover + wheat bran based medium. Based on the stained bands of the first column, the correspondent gel bands on the second column of the same sample were cut as narrow as possible and these cuts were separately incubated in *p*NPG for 10 min. Actually, bands Glu2,Glu3,Glu4,Glu7,CW2,CW4,CW5 changed to yellow. That proved existence of β-glucosidase activity. Among these stained bands, colors of band Glu7 and CW2 were the most visible. The position of band Glu2 at the gel corresponded to that of band CW2, band Glu3 matched with band CW4, and band Glu4 was corresponding to band CW5. Corresponding band of Glu7 was not found at the CW gel. Band CW7 was cut out of CW gel based on the position of band Glu7 on the assumption that the same β-glucosidases would be produced by *F. proliferatum* that grows in different carbon sources. The cut was treated for activity staining but no change of color was observed. It indicated that the cut did not contain any β-glucosidase. Subsequently, the bands with β-glucosidase activity on the second column were used as markers to cut the corresponding bands out of the third and fourth column of the same sample as narrow as possible. The cuts were soaked in acetate buffer (0.05 M, pH5.0) to recover the protein of β-glucosidase containing in the gel and treated with SDS-PAGE and then with silver staining. Figure 5-b indicates the results of the SDS-PAGE. However, the amount of proteins existing in bands Glu2,Glu3,Glu4,CW4 and CW5 was too low to be visible after the SDS-PAGE. In all, at least four different β-glucosidases were produced by *F. proliferatum* growing in glucose based medium and at least three different β-glucosidases were produced by *F. proliferatum* growing in medium of corn stover + whea bran. β-Glucosidase with the molecular weight of approximate 46 was produced in glucose based medium only. Therefore, we came to a conclusion that different β-glucosidases can be produced by that grows in different carbon based mediums.

### Partial purification of β-glucosidase

**Figure 6:**
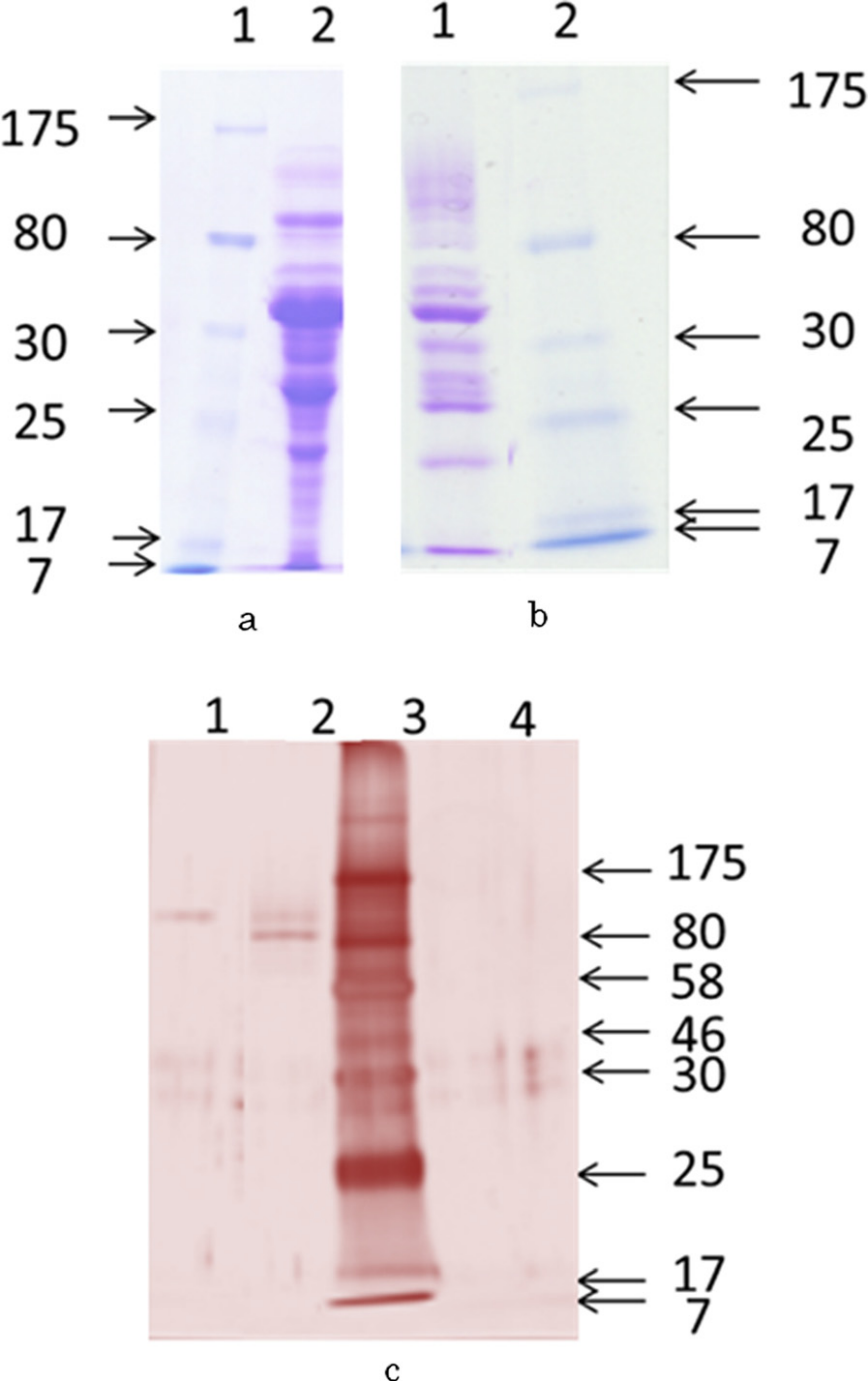
**SDS-PAGE analysis (10% polyacrylamide) of protein sample from each step of the partial purification. **a-(1), molecular weight markers; a-(2), crude protein extract; b-(1), the active peak from DEAE sepharose CL-6B; b-(2), molecular weight markers; c-1, the protein from CW2 band of the modified zymogram; c-(2), the protein from the active peak of Superdex75; c-(3) molecular weight markers; c-(4), loading buffer only. The protein showed on A and B was stained with CBB R-250. The protein showed on C was with silver staining.

The partial purification process was summarized in Table 4. In the initial step of purification with ammonium sulfate fractionation, about 70% of total β-glucosidase activities could be recovered in the fraction of 50–70% ammonium sulfate saturation with a purification of 3.3 times. In the second step, ion-exchange chromatography on DEAE-Sepharose CL-6B was performed using five concentration of sodium chloride for elution. In this step, greater purity was realized since most of the contaminating protein was removed. β-glucosidase was eluted from the ion exchanger at the sodium chloride concentration of 0.15 M, as one broad peak. About 32% of total β-glucosidase activities could be recovered. Accordingly, β-glucosidase was purified 9.2 times. In the third step, active fraction (0.15 M NaCl) gained from DEAE-Sepharose CL-6B was applied on Superdex 75 column. About 16% of total β-glucosidase activities could be recovered. As a result, β-glucosidase was purified 18.0 times. After all these steps, we got β-glucosidase that had a specific activity of 287.7 U/mg based on *p*NPG and 6 400 U/mg based on cellobiose. The results pointed out that β-glucosidase produced by *F. proliferatum* that grows in corn stover + wheat bran based medium has high catalytic efficiency (Table 5). There were two major bands on the SDS-PAGE of the active peak from Superdex 75. Compared the location band of CW2 that came from the modified zymogram and active peak from superdex75 (Figure 6-c), we can get the conclusion that the band on the top of lane 2 on Figure 6-c is the β-glucosidase we need to purify.

**Table 4:** **Summary of the purification steps of the β-glucosidase produced by *F. proliferatum *growing in corn stover + wheat bran based medium**

**Step**	**Total activity (U)**	**Total protein (mg)**	**Specific activity (U/mg)**	**Purification factor**	**Recovery (%)**
Crude extract	150	9.38	16.0	1.0	100
(NH4)2SO4	106	1.98	53.5	3.3	70.4
DEAE Sepharose CL-6B	48.6	0.33	148	9.2	32.3
Superdex75	23.8	0.08	288	18	15.8

**Table 5:** Specific activity of purified β-glucosidase from various sources

**Strain**	**Specific activity (U/mg)**	**Reference**
*Rhizomucor miehei *(NRRL 5282)	62	(Krisch et al. 2012)
*Candida peltata *(NRRL Y-6888)	108	(Saha and Bothast 1996)
*Daldinia eschscholzii*	78	(Karnchanatat et al. 2007)
*Stachybotrys microspora*	20	(Saibi and Gargouri 2011)
Thymepkilic anaerobic bacterium	149	(Patchett et al. 1987)
*Aspergillus niger* (NIAB 280)	42	(Rashid and Siddiqui 1997)
*Xylaria regalis*	23	(Wei et al. 1996)
*Trichoderma sp.*	214	(Fadda et al. 1994)
*Aspergillus niger *(CCRC 31494)	199	(Yan and Lin 1997).
*Fusarium proliferatum *(NBRC 109045)	288*	This study

*partially purified.

## Discussion

Cellobiose was considered as an inducer of cellulase which includes β-glucosidases (Mandels and Reese 1957). However, the amount of β-glucosidases when *F. proliferatum* grew in cellobiose based medium was less than that in corn stover + wheat bran based medium. When compared the yield of β-glucosidases in cellobiose based medium with that in corn stover + wheat bran based medium, *F. proliferatum* grew in cellobiose faster than that in corn stover + wheat bran (data not shown). This proved that cellobiose is an excellent growth substance for and is rapidly consumed, whereas corn stover + wheat bran is a relatively poor growth substance and is slowly consumed. The same phenomenon was observed by (Mandels and Reese 1960). They held the opinion that the inhibitory effect of cellobiose on β-glucosidases production seems to be related to rapid metabolism of the cellobiose.

Wheat bran that contains significant quantities of starch, protein and so on is a rich source of nutrients and could promote growth and enzyme production of fungus. Corn stover that is mainly composed of lignocellulose is a very common and cost-free agricultural product. Supplementation of the mixture of wheat bran and corn stover resulted in a significant increase in β-glucosidases activity when compared to individual application. The likely reasons for the result were that wheat bran provided *F. proliferatum* with adequate nutrition at the early growth stage and made the strain grow fast. After nutrition contained in wheat bran ran out, *F. proliferatum* could hardly get required nutrition from corn stover and became starve and then produced a huge amount of β-glucosidases.

*F. proliferatum* did not produce β-glucosidases in glucose or cellobiose based medium without addition of urea (Table 2). And it has been proved that there was no direct relationship between addition of urea and halting reduction in the pH of glucose or cellobiose based medium. Emergence of the phenomenon prompted us to ponder a problem that how the addition of urea contributed to β-glucosidase production in glucose or cellobiose based medium. This is probably due to the metabolites produced by *F. proliferatum* growing in glucose or cellobiose based medium or the derivatives of the components containing in the glucose or cellobiose based medium. The metabolites or derivatives produced by the fungus would reduce the pH of the glucose or cellobiose based medium. Low pH of the glucose or cellobiose based medium, in turn, cut the production of β-glucosidases. But addition of urea at the beginning of cultivation can cut the production of the metabolites or derivatives. That, in turn, halted reduction in pH of glucose or cellobiose based medium. However, adding urea to the glucose or cellobiose based medium after 8-day cultivation cannot damage the metabolites or derivatives produced in large quantities during incubation. In this case β-glucosidases still cannot be produced by *F. proliferatum* even addition of urea. The possible reasons for slight decrease in pH of the corn stover + wheat bran based medium are because the metabolites or derivatives were not produced by *F. proliferatum*, or only tiny amount of the metabolites or derivatives was produced. Therefore, urea addition did not affect the production of β-glucosidases produced by *F. proliferatum* growing in corn stover + wheat bran based medium significantly.

β-Glucosidases produced by *F. proliferatum* in different carbon sources based mediums expressed varied glucose tolerance (Figure 3). (Isorna et al. 2007) purified a β-glucosidase, named as BglB, produced by *P. polymyxa* and obtained the crystallographic structure of the BglB with glucose. In this structure, the ring of glucose resided in the active site, through the interactions with nine amino acids of BglB. Of the nine residues, seven were involved in intermediate binding to glucose directly, while the other two, Trp412 and His181, indirectly binding to glucose. The seven directly interacting residues were found to conserve among different β-glucosidases belonging to GH1, whereas Trp412 and His181 in BglB are fairly variable. The two variable residues were assumed to play important roles in glucose tolerance. It has been proved that the 184^th^ residue of β-glucosidase BglB plays an important role in glucose tolerance (Liu et al. 2011). Glucose acts as an inhibitor by competing with the substrate in binding to the enzyme (Fang et al. 2010). But the mechanism of β-glucosidase tolerance to glucose is still unclear. Presumably the cause is that there are variable special residuals on active site of β-glucosidases by *F. proliferatum* in different carbon sources based mediums. These residuals are not only the binding site of glucose but the binding site of the substrate, so changes of the special residuals cause difference in degree of bond to glucose. That, in turn, makes difference in level of tolerance to glucose.

We reported the modified zymogram method that is a combination of the technology zymogram, gel purification and SDS-PAGE to prove that *F. proliferatum* could express varied β-glucosidases in respond to varied carbon sources. The approach described in the paper overcomes the disadvantages of applying crude enzyme on zymogram and combines effective isolation with identification assay.

## Competing interests

The authors declare that they have no competing interests.
